# Immuno-inflammatory axis of menopausal dry eye: from hormonal fluctuations to ocular surface dysregulation

**DOI:** 10.3389/fimmu.2026.1732320

**Published:** 2026-03-27

**Authors:** Zi-ting Tian, Chun-ying Jing, Hong-bo Liu, Xiao-kang Jia, Hong-yun Xu, Yan-yang Pang

**Affiliations:** 1 Department of Traditional Chinese Medicine External Medicine, College of Traditional Chinese Medicine, Hainan Medical University, Haikou, China; 2 Department of Traditional Chinese Medicine, Hainan Women and Children’s Medical Center, Haikou, China

**Keywords:** dry eye disease, immuno-inflammatory axis, inflammation, mechanism, menopause, review

## Abstract

Menopause is a significant risk factor for dry eye disease (DED), conventionally explained by estrogen deficiency and diminished tear production. Recent evidence, however, highlights the pivotal role of immune-inflammatory mechanisms in its development. This mini-review aims to integrate contemporary insights into the immuno-inflammatory pathways active in menopausal DED, clarifying how variations in sex hormones initiate and perpetuate immune dysregulation within the ocular surface microenvironment. We propose that menopausal DED is not solely a disorder of inadequate lubrication but rather a chronic immune-mediated inflammatory state, propelled by hormonal shifts. Its pathology is defined by a self-sustaining cycle of T cell-led inflammation, loss of goblet cells, and neurosensory dysfunction, which together exacerbate ocular surface impairment and clinical symptoms.

## 1 Introduction

Menopausal dry eye disease (DED) is a common yet underrecognized condition with a high prevalence among middle-aged and elderly women worldwide (1, 2). Epidemiological studies indicate that the incidence of DED is significantly higher in perimenopausal and postmenopausal women compared to males of the same age, substantially impacting visual function, daily activities, and emotional well-being, thereby representing a considerable public health burden (3, 4). Traditionally, menopausal DED was attributed simply to declining sex hormone levels—particularly estrogen—believed to directly reduce tear secretion and ocular surface lubrication (5). However, as research has advanced, this “hormone-deficient dryness” paradigm has revealed notable limitations: clinical evidence shows that estrogen replacement therapy alone offers limited and sometimes controversial symptomatic improvement in some patients, suggesting that the pathogenesis may involve more complex pathophysiological mechanisms (5, 6).

Increasing evidence indicates that menopausal DED should be interpreted within the broader framework of a neuro–endocrine–immune regulatory network (7). Sex hormones not only regulate ocular surface physiology but also exert profound immunomodulatory effects across multiple immune pathways (7, 8). Receptors for estrogen and androgens are widely expressed in immune cells—including T lymphocytes, dendritic cells, and macrophages—allowing systemic hormonal fluctuations to influence immune signaling directly (7, 9). Consequently, the hormonal imbalance characteristic of menopause can reshape cytokine expression profiles, promote immune cell activation, and weaken mechanisms of immune tolerance (10–12). Through these processes, endocrine alterations become mechanistically coupled to local immune dysregulation at the ocular surface (13). This endocrine–immune interaction provides a critical biological bridge linking systemic hormonal changes with the chronic inflammatory responses observed in menopausal DED, thereby reframing the condition not simply as a tear-deficiency disorder but as a consequence of disrupted immune–endocrine signaling (13).

In recent years, a paradigm shift has emerged in the understanding of menopausal DED—transitioning from a hormone-centric view toward recognizing it as an “immune-inflammatory disease.” This shift is supported by extensive clinical and basic research evidence (14, 15). During menopause, the decline in ovarian function not only reduces estrogen levels but also causes fluctuations in other hormones such as androgens and progesterone (14). These endocrine changes can directly or indirectly affect immune homeostasis on the ocular surface and in the lacrimal glands, promoting the activation of local inflammatory responses (14). Specifically, decreased sex hormone levels can upregulate the expression of inflammatory cytokines (e.g., interleukin-1 beta (IL-1β), tumor necrosis factor-alpha (TNF-α), matrix metalloproteinases (MMPs)) in the lacrimal gland, while simultaneously impairing the epithelial barrier function and stability of the tear film (15, 16). Furthermore, hormonal fluctuations may enhance immune cell infiltration and activation through signaling pathways such as nuclear factor kappa-B (NF-κB), leading to a chronic inflammatory environment primarily mediated by T cells (17–19). This “immune-inflammatory axis” not operates independently of traditional secretory deficiency mechanisms but may also synergize with them to collectively drive disease progression (17, 18).

Against this background, this review aims to systematically elucidate the molecular and cellular mechanisms underlying the transition from hormonal fluctuations to immune dysregulation in menopausal DED. We will focus on how sex hormones modulate innate and adaptive immune responses via receptor-mediated pathways and analyze the interactions among key cell types—such as goblet cells, corneal epithelial cells, dendritic cells, and T lymphocytes—in disease pathogenesis. Finally, based on these mechanisms, we will discuss potential targeted treatment strategies, including the application of topical anti-inflammatory agents, cytokine inhibitors, and immunomodulators, to provide a theoretical foundation and future directions for clinical management.

## 2 The core driver: hormonal fluctuations in menopause

The pathogenesis of menopausal DED is fundamentally driven by significant fluctuations and imbalances in sex hormone levels, rather than a simple deficiency of a single hormone (5, 20, 21). Estrogens, androgens, and other hormones together form a complex regulatory network that directly influences the structural and functional integrity of the ocular surface (20, 21).

### 2.1 The dual role of estrogen

Estrogen plays a multifaceted and complex role in maintaining ocular surface homeostasis. On one hand, it exerts well-established protective effects: estrogen receptors are widely distributed in the lacrimal glands, meibomian glands, cornea, and conjunctival epithelial cells (10, 14). Activation of these receptors promotes secretory function of the lacrimal gland, maintains normal goblet cell density and mucin secretion in the conjunctiva, thereby stabilizing the tear film structure (14, 15, 22). Simultaneously, it regulates lipid secretion from the meibomian glands and helps preserve corneal epithelial barrier integrity (14). Furthermore, estrogen demonstrates anti-inflammatory properties, such as inhibiting the production of various pro-inflammatory cytokines through antagonism of classical inflammatory signaling pathways like NF-κB, thereby maintaining immune homeostasis under physiological conditions (10, 14, 15, 22).

However, the actions of estrogen are not straightforward and exhibit significant tissue specificity and receptor subtype selectivity. Different receptor subtypes (e.g., ERα and ERβ) may mediate distinct biological effects in different cell types, explaining why systemic or local estrogen interventions often yield inconsistent or even contradictory results in clinical studies (21, 23–25). Thus, although the decline in estrogen during menopause is associated with DED, its impact should be considered within the broader context of the hormonal network.

### 2.2 The critical role of androgens

Androgens, particularly testosterone, play a more crucial role than previously recognized in regulating the function of the lacrimal and meibomian glands (14, 21). Both glands express androgen receptors, and androgens activate these receptors to directly stimulate the secretion of lipid and aqueous components from glandular epithelial cells, which is essential for maintaining the quantity and quality of the tear film (14, 26). Clinical observations have shown that the decline in androgen levels during perimenopause closely correlates with the onset of dry eye symptoms (12, 27). Some studies even suggest that the decrease in androgens may be a more direct and potent driver than estrogen withdrawal, potentially explaining why the prevalence of DED is generally lower in men than in women of the same age—androgen decline in men typically occurs more gradually with aging (14, 26).

### 2.3 Network effects of hormonal interactions

The origin of menopausal DED is not the deficiency of a single hormone but rather the disruption of the balance between estrogen and androgen (12, 20). This imbalance collectively fosters a pro-inflammatory microenvironment. This imbalance collectively fosters a pro-inflammatory microenvironment within the lacrimal functional unit and the ocular surface. For instance, the reduction in estrogen diminishes its inherent anti-inflammatory effects, while the decline in androgen directly impairs glandular secretory function (20, 28).

Beyond their direct effects on glandular function, sex hormones play a crucial role in regulating immune homeostasis. Estrogen and androgen signaling pathways influence the differentiation, activation, and functional balance of multiple immune cell subsets, including T helper 1 (Th1), T helper 17 (Th17), and regulatory T (Treg) cells (14). During menopause, the decline and imbalance of these hormones can disrupt this regulatory network, shifting immune responses toward a pro-inflammatory phenotype (14, 17). This endocrine-driven immune modulation enhances the production of inflammatory cytokines and promotes immune cell recruitment to ocular tissues, including the lacrimal glands and ocular surface (18, 29). As a consequence, the local immune microenvironment becomes increasingly permissive to inflammatory activation and tissue damage (14, 15, 18).

Together, these endocrine and immune alterations render the ocular surface more susceptible to immune cell infiltration and inflammatory mediators. This hormone imbalance triggers a cascade of reactions, ultimately leading to tear film instability, ocular surface epithelial damage, and neurosensory abnormalities, marking the transition of the disease from a purely secretory-deficient state to a chronic immune-inflammatory condition (12, 20, 28). Therefore, understanding the endocrine–immune regulatory network underlying menopausal hormonal imbalance is essential for elucidating disease pathogenesis and for developing targeted therapeutic strategies that address the hormonal regulatory axis (**Table 1**).

**Table 1:** **Key hormones, immune cells, and cytokines involved in menopausal dry eye disease**.

Category	Key factors	Alterations	Major functions and pathogenic roles	References
Hormones	Estrogen	Significant decline	Promotes lacrimal gland secretion, maintains goblet cell density and mucin secretion, stabilizes tear film; exerts anti-inflammatory effects (e.g., NF-κB inhibition). Decline leads to tear film instability and enhanced inflammation.	Suzuki et al. (10); Gorimanipalli et al. (14); Hat et al. (21)
	Androgen (e.g., testosterone)	Decline	Stimulates lipid and aqueous secretion from meibomian and lacrimal glands; maintains tear film quality. Decline impairs glandular secretion and promotes inflammation.	Krenzer et al. (13); Wang et al. (26)
	Progesterone	Fluctuation	Together with estrogen and androgens, regulates ocular surface immune homeostasis. Fluctuations further exacerbate immune imbalance.	Sullivan et al. (8); Gorimanipalli et al. (14)
Immune cells	Dendritic cells	Increased maturation and antigen-presenting capacity	Act as “sentinels” of innate immunity; initiate adaptive immune responses.	Ortiz et al. (30); Stevenson et al. (31)
	Macrophages	Activated	Secrete IL-1, IL-6, TNF-α; amplify local inflammation.	Kaur et al. (32); de Souza et al. (33)
	CD4+ T cells (Th1, Th17)	Increased activation and infiltration	Th1 produces IFN-γ; Th17 produces IL-17, amplifying inflammation and recruiting neutrophils.	El Annan et al. (34); Chauhan et al. (35)
	Treg cells	Reduced number and suppressive function	Weakened immunosuppressive effect; Th17/Treg imbalance sustains chronic inflammation.	Chauhan et al. (35); Alam et al. (36)
Cytokines	IL-1β	Upregulated	Induces inflammation and disrupts epithelial barrier.	VanDerMeid et al. (37); Yamaguchi (38)
	IL-6	Upregulated	Promotes inflammation and Th17 differentiation.	Kaur et al. (32); Zhang et al. (39)
	TNF-α	Upregulated	Induces goblet cell apoptosis, impairs lacrimal gland function, and promotes neuroinflammation.	Kelagere et al. (40); Yamaguchi (38)
	IL-17	Markedly increased	Secreted by Th17 cells; recruits neutrophils, amplifies inflammation, and disrupts epithelial barrier.	Alam et al. (36); De Paiva et al. (41)
	MMP-9	Increased activity	Degrades basement membrane and tight junction proteins, increases corneal epithelial permeability, and impairs barrier function.	Pflugfelder et al. (42); Pflugfelder et al. (43)
Effector cells/tissues	Goblet cells	Reduced	Decreased mucin secretion, impaired tear film adhesion.	Pflugfelder et al. (44); De Paiva et al. (41)
	Lacrimal gland epithelial cells	Functional impairment	Reduced secretion; chronic inflammation causes fibrosis of glandular tissue.	de Souza et al. (33); Pflugfelder et al. (44)
	Corneal nerve fibers	Neuroinflammation aggravated	Leads to neuropathic pain, foreign body sensation, and dryness.	Vereertbrugghen et al. (45); Fakih et al. (46)

NF-κB, Nuclear Factor kappa-light-chain-enhancer of activated B cells; Th1, T helper type 1 cells; Th17, T helper type 17 cells; Treg, Regulatory T cells; IFN-γ, Interferon-gamma; IL-1β, Interleukin-1 beta; IL-6, Interleukin-6; TNF-α, Tumor Necrosis Factor-alpha; IL-17, Interleukin-17; MMP-9, Matrix Metalloproteinase-9.

## 3 The central link: activation of the immuno-inflammatory axis

**Figure 1:**
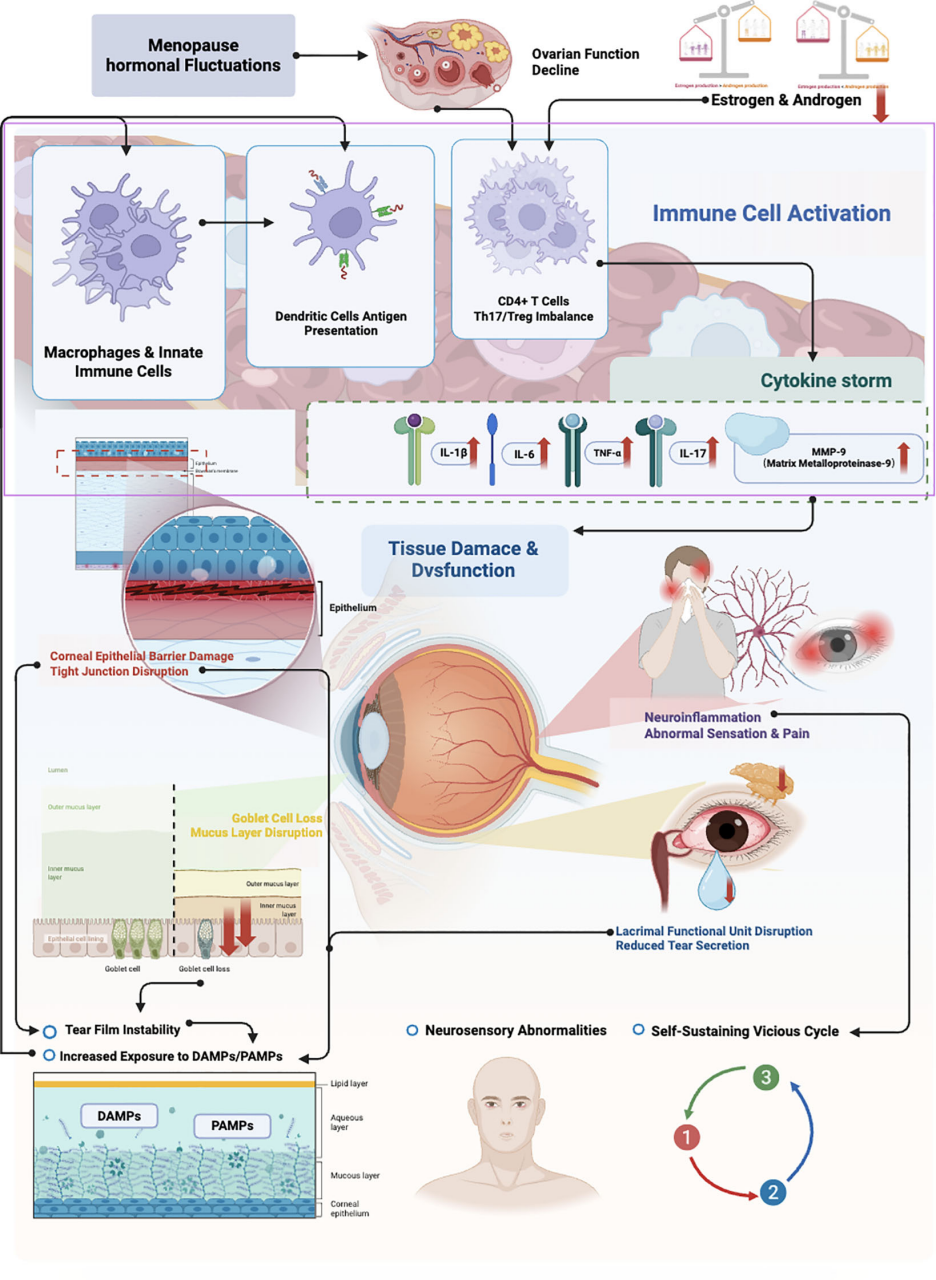
Core mechanism of the immuno-inflammatory axis in menopausal dry eye disease.

The immuno-inflammatory response triggered by menopausal hormonal fluctuations represents a core pathological mechanism driving the onset and progression of DED (**Figure 1**). This process involves the sequential activation and mutual amplification of innate and adaptive immunity, ultimately leading to the loss of ocular surface homeostasis and functional impairment (30, 32, 47).

### 3.1 Dysregulation of the innate immune system

Changes in the hormonal milieu directly predispose the ocular surface and lacrimal glands to a pro-inflammatory state. Resident innate immune cells, such as macrophages and corneal epithelial cells, become activated and release substantial quantities of pro-inflammatory cytokines, including interleukin-1, interleukin-6, and TNF-α (33, 42). These mediators create a “storm” of inflammation that not only directly damages tissue function but also recruits and activates additional immune cells (33, 42). Concurrently, the expression and activity of MMPs, such as MMP-9, are significantly upregulated. MMP-9 degrades the basement membrane and intercellular junctional proteins within the corneal epithelium, compromising epithelial barrier integrity, increasing permeability, and promoting conjunctival remodeling—all of which exacerbate tear film instability (42, 43). Moreover, dendritic cells, which serve as critical “sentinels” linking innate and adaptive immunity, exhibit enhanced maturation and antigen-presenting capacity in the menopausal microenvironment (30, 42) (**Table 1**). This functional shift disrupts immune tolerance and provides a necessary foundation for T cell activation and subsequent antigen-specific immune responses.

### 3.2 Ignition of adaptive immunity: the central role of T cells

Sustained innate immune signaling leads to robust activation of the adaptive immune system, in which CD4^+^ T lymphocytes play a pivotal role (34). A key feature is the disruption of the balance between Th17 cells and Tregs (35) (**Table 1**). Within the menopausal inflammatory environment, the Th17 cell subset expands significantly and produces large amounts of the effector cytokine interleukin-17 (IL-17) (36). IL-17 potently recruits neutrophils and further stimulates epithelial cells to produce additional inflammatory cytokines and chemokines, creating a positive feedback loop that amplifies and sustains inflammation (35, 39). Simultaneously, the number and immunosuppressive function of Tregs are diminished, reducing their ability to negatively regulate the inflammatory response (36, 48). This Th17/Treg imbalance is a crucial mechanism underlying the persistence of chronic inflammation. Activated CD4+ T cells (including Th1 and Th17 subsets) extensively infiltrate the ocular surface and lacrimal glands, where they remain in a persistent state of activation (34). There is also evidence suggesting that prolonged inflammation may expose self-antigens, break immune tolerance, and induce an autoimmune-like response—a process further exacerbated by systemic immunosenescence and declined immune surveillance associated with menopause (34, 35).

### 3.3 Damage at the effector level

The persistent immuno-inflammatory response ultimately inflicts structural and functional damage on effector tissues and cells of the ocular surface (**Table 1**). Goblet cells, which are highly susceptible to inflammatory mediators, undergo apoptosis induced directly by cytokines such as IL-17 and TNF-α (36, 44). Their widespread loss leads to deficient mucin secretion, severely compromising tear film adhesion and stability. In the lacrimal gland, chronic inflammatory cell infiltration causes destruction of acinar and ductal structures, resulting in secretory cell dysfunction and eventual replacement by fibrous tissue—a process termed “lacrimal functional unit disruption” (40, 44). Furthermore, inflammatory factors directly affect corneal nerve fibers, inducing neuroinflammation (44, 45). This not only causes neuropathic pain and dysesthetic sensations (such as dryness, burning, and foreign body sensation) but also further deteriorates the ocular surface microenvironment through neuro-immune-endocrine interactions, forming a self-sustaining vicious cycle (36, 44, 45).

## 4 The vicious cycle: from inflammation to ocular surface dysregulation

**Figure 2:**
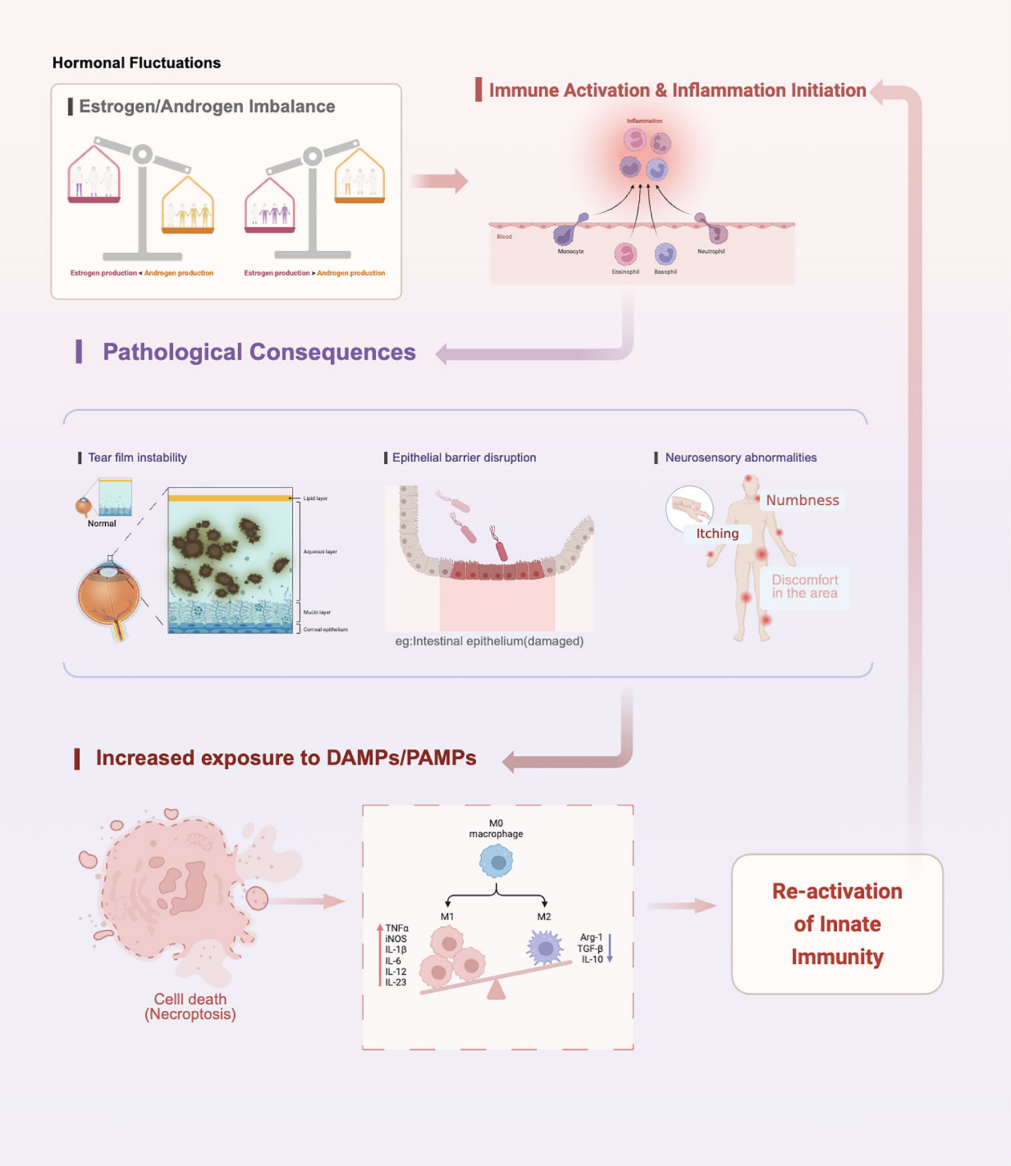
The self-sustaining vicious cycle of menopausal dry eye disease.

In menopausal DED, hormonal imbalance serves as the initiating trigger that activates a downstream inflammatory vicious cycle on the ocular surface. Central to this cycle is a positive feedback loop between immune-inflammatory activation and ocular surface tissue damage, ultimately leading to chronicity and treatment resistance (31, 49) (**Figure 2**). In contrast to other forms of DED, this pathological cascade is fundamentally driven by endocrine disturbances associated with menopause—most notably the decline and altered balance of estrogen and androgens (12). Such hormonal alterations increase the susceptibility of ocular surface tissues and lacrimal functional units to immune activation, thereby establishing the initial conditions for persistent inflammatory responses (12, 50).

The cycle is initiated by fluctuations in sex hormone levels during menopause, particularly the decline and altered ratio of estrogen and androgens (14, 28). This endocrine shift directly promotes a pro-inflammatory microenvironment in the ocular surface and lacrimal functional unit, serving as the initial trigger for immune activation (10, 37) (**Table 1**). This is followed by immune activation and the initiation of inflammation: innate immune cells (e.g., dendritic cells and macrophages) are mobilized, pro-inflammatory cytokines (such as TNF-α, IL-1β, and interleukin-6) are released in large quantities, and the activity of matrix metalloproteinases (e.g., MMP-9) is upregulated (10, 28, 37). Concurrently, adaptive immune responses—especially Th17-mediated inflammation—are significantly enhanced (14, 37).

These immuno-inflammatory events lead to three major pathological consequences: (1)Severe tear film instability due to abnormal lipid and mucin secretion, accelerating tear evaporation (36, 51); (2) Disruption of the ocular surface epithelial barrier, compromising corneal and conjunctival epithelial tight junctions and increasing permeability (38, 41, 52); (3)Neurosensory abnormalities, where inflammatory cytokines induce neuroinflammation, resulting in pain, hypersensitivity, and dysesthesia (46, 53).

These damaging outcomes further expose more damage-associated molecular patterns (DAMPs) (54, 55). Damaged or apoptotic epithelial cells release DAMPs (e.g., HMGB1, heat shock proteins), while tear film instability and barrier dysfunction also increase exposure to exogenous pathogen-associated molecular patterns, such as bacterial lipopolysaccharides (54–57). These molecules are recognized by pattern recognition receptors on local immune cells (e.g., dendritic cells, epithelial cells), thereby reactivating and amplifying the innate immune response, particularly through signaling pathways such as NF-κB, leading to further production of inflammatory mediators (55–57).

As a result, the inflammatory loop is re-triggered and continuously reinforced: each round of immune response causes more severe ocular surface damage, which in turn releases additional danger signals that further stimulate the immune system (58–60). This creates a self-driven cyclical pathological process (59, 61). Without therapeutic intervention to break this cycle, inflammation and tissue damage will continue to mutually reinforce each other, ultimately leading to chronicity and reduced responsiveness to conventional treatments (58, 60).

## 5 Clinical implications and therapeutic perspectives

The in-depth understanding of the pathogenesis of menopausal DED, particularly its core immuno-inflammatory axis, is profoundly reshaping clinical management strategies and providing a theoretical foundation for the development of novel therapeutic approaches.

### 5.1 Shift in diagnostic thinking

Traditional diagnosis has primarily relied on symptomatology and basic metrics such as tear secretion volume. However, given the immuno-inflammatory nature of the disease, future diagnostic workflows should incorporate an assessment of inflammatory status (62, 63). For example, point-of-care testing for ocular surface inflammatory biomarkers (e.g., rapid immunoassays for MMP-9) has emerged as a feasible auxiliary tool (62, 64, 65). Although biomarkers such as MMP-9 are frequently used to assess inflammatory activity in DED, they are not specific to the menopausal subtype (66). Instead, they primarily reflect the presence and intensity of ocular surface inflammation and should therefore be interpreted as indicators of generalized inflammatory processes rather than disease-specific markers (67). Nevertheless, the detection of elevated inflammatory biomarkers may still provide important clinical value. A positive MMP-9 result can objectively confirms the presence of active ocular surface inflammation, thereby supporting the identification of patients who may benefit from targeted anti-inflammatory therapies. In this context, biomarker-guided assessment helps shift the diagnostic and therapeutic paradigm from a purely symptom-oriented approach toward one that addresses the underlying inflammatory mechanisms of the disease (62, 63, 65).

### 5.2 Evaluation of current anti-inflammatory therapies

Currently, targeting the immuno-inflammatory component is central to managing moderate-to-severe cases. Topical corticosteroids non-specifically inhibit multiple inflammatory pathways and act rapidly, making them suitable for short-term control of acute inflammatory flares, though long-term use is limited by side effects (68, 69). Calcineurin inhibitors (e.g., cyclosporine A and tacrolimus) offer a safer option for long-term anti-inflammatory management (68). By inhibiting T-cell activation and cytokine production, they modulate the immune response precisely (68, 70). Multiple studies have confirmed their efficacy in improving ocular surface inflammatory markers and patient symptoms, particularly for maintenance therapy in chronic cases (68, 69, 71).

### 5.3 Emerging and future targeted strategies

As disease pathways are elucidated in greater detail, increasingly precise therapeutic strategies are emerging for the management of menopausal DED. In contrast to general anti-inflammatory therapies commonly used for DED, menopausal dry eye may benefit more from interventions that directly address the underlying hormonal imbalance associated with menopause (72). Restoring a physiological hormonal microenvironment on the ocular surface may therefore represent a complementary therapeutic strategy alongside conventional anti-inflammatory approaches (72). To achieve this while minimizing systemic adverse effects, current research is increasingly focusing on localized hormone-based therapies—particularly topical formulations containing androgens or estrogens—which aim to correct local hormonal deficiencies more precisely and potentially offer a safer alternative to systemic hormone replacement (73).

Biologics: Monoclonal antibodies against key inflammatory cytokines (e.g., anti-TNF-α, anti-IL-17A) are well-established in treating autoimmune conditions (36, 74). Their potential use in severe, refractory menopausal dry eye is under investigation. However, the risk-benefit profile of systemic administration requires careful evaluation, and the development of local delivery systems may be a critical direction.

Neuro-immunomodulators: This class of agents aims to simultaneously address both core issues of pain and inflammation. By targeting interaction signals between corneal nerve endings and immune cells (e.g., via neuropeptide inhibitors), they may alleviate pain and dysesthesia while indirectly reducing neurogenic inflammation (75, 76).

Precision hormone intervention: Conventional systemic hormone replacement therapy has shown inconsistent effects on dry eye (12). New strategies favor topical application of low-dose hormones (e.g., estrogen eye drops) or the use of tissue-selective estrogen receptor modulators to achieve desirable hormonal effects locally while minimizing systemic side effects (77, 78).

Microbiome modulation: Growing evidence suggests that dysbiosis of the gut and ocular surface microbiota can influence dry eye development by modulating systemic and local immunity (79–81). Thus, interventions such as probiotics, prebiotics, or dietary modifications to restore microbial balance—and indirectly suppress ocular surface inflammation—represent a promising new avenue for treatment.

## 6 Summary and future directions

In summary, menopausal DED is more accurately defined as an immune-inflammatory condition driven by hormonal changes. Its pathological core lies in a self-perpetuating cycle of chronic inflammation, primarily mediated by an imbalance between Th17 and Treg cells, which sustains ocular surface damage and dysfunction.

Targeting the immuno-inflammatory axis represents a fundamental therapeutic strategy for managing this condition. Moving beyond conventional lubrication-based approaches, interventions that specifically address immune dysregulation—such as immunomodulators and targeted biologic agents—are essential for achieving sustained disease control and restoring ocular surface homeostasis.

Future research should focus on several key areas: first, the identification of specific biomarkers capable of stratifying patients and predicting treatment responses; second, the conduct of rigorous clinical trials evaluating novel immunotherapeutic agents in well-defined menopausal populations; and finally, deeper investigation into the complex interactions within the neuro-immune-endocrine network on the ocular surface, which may reveal new mechanistic insights and therapeutic opportunities for breaking the cycle of chronic inflammation.
